# Deep divergence and rapid evolutionary rates in gut-associated Acetobacteraceae of ants

**DOI:** 10.1186/s12866-016-0721-8

**Published:** 2016-07-11

**Authors:** Bryan P. Brown, Jennifer J. Wernegreen

**Affiliations:** Nicholas School of the Environment, Duke University, Box 3382, Durham, NC 27708 USA; Center for Genomic and Computational Biology, Duke University, Box 3382, Durham, NC 27708 USA

**Keywords:** Gut microbiota, Phylogenetic diversification, Insects, Eusociality, Acetic acid bacteria

## Abstract

**Background:**

Symbiotic associations between gut microbiota and their animal hosts shape the evolutionary trajectories of both partners. The genomic consequences of these relationships are significantly influenced by a variety of factors, including niche localization, interaction potential, and symbiont transmission mode. In eusocial insect hosts, socially transmitted gut microbiota may represent an intermediate point between free living or environmentally acquired bacteria and those with strict host association and maternal transmission.

**Results:**

We characterized the bacterial communities associated with an abundant ant species, *Camponotus chromaiodes*. While many bacteria had sporadic distributions, some taxa were abundant and persistent within and across ant colonies. Specially, two Acetobacteraceae operational taxonomic units (OTUs; referred to as AAB1 and AAB2) were abundant and widespread across host samples. Dissection experiments confirmed that AAB1 and AAB2 occur in *C. chromaiodes* gut tracts. We explored the distribution and evolution of these Acetobacteraceae OTUs in more depth. We found that *Camponotus* hosts representing different species and geographical regions possess close relatives of the Acetobacteraceae OTUs detected in *C. chromaiodes*. Phylogenetic analysis revealed that AAB1 and AAB2 join other ant associates in a monophyletic clade. This clade consists of Acetobacteraceae from three ant tribes, including a third, basal lineage associated with Attine ants. This ant-specific AAB clade exhibits a significant acceleration of substitution rates at the 16S rDNA gene and elevated AT content. Substitutions along 16S rRNA in AAB1 and AAB2 result in ~10 % reduction in the predicted rRNA stability.

**Conclusions:**

Combined, these patterns in *Camponotus*-associated Acetobacteraceae resemble those found in cospeciating gut associates that are both socially and maternally transmitted. These associates may represent an intermediate point along an evolutionary trajectory manifest most extremely in symbionts with strict maternal transmission. Collectively, these results suggest that Acetobacteraceae may be a frequent and persistent gut associate in *Camponotus* species and perhaps other ant groups, and that its evolution is strongly impacted by this host association.

**Electronic supplementary material:**

The online version of this article (doi:10.1186/s12866-016-0721-8) contains supplementary material, which is available to authorized users.

## Background

Associations between gut bacteria and their hosts have had profound impacts on the evolutionary trajectories of both partners [1]. These interactions have facilitated the success of many animal groups by providing key benefits to the host, such as contributions to host nutrition in termites [2], immune defense in moths [3], kin recognition in *Drosophila* [4], and toxin production in the antlion [5]. While fitness benefits for bacterial mutualists remain less obvious, adaptation to the gut environment, in some cases of a particular host species, has significant impacts on the evolution of gut associates.

Among bacteria, inhabiting an animal gut can have a range of genomic consequences. These can include general adaptations that favor colonization of the gut environment and long-term signatures of coevolution with a specific host. In murine models, increased bacterial mutation rates have been demonstrated to facilitate colonization of the gut niche by expediting adaptation [6]. Likewise, the genomes of acetic acid bacteria, a family of known insect symbionts [7], encode cytochrome bo_3_ ubiquinol oxidase, which may facilitate survival across both normoxic and micro-oxic conditions encountered in the gut ecosystem [8–10]. Genomic studies of bee associates have reported syntrophic networks among bacterial mutualists and the presence of many genes that may facilitate gut colonization and cell-cell interactions [11]. These traits, as well as vertical transmission, may mediate host specificity [11]. Furthermore, genetic characteristics of persistent gut associates can include AT-biased nucleotide composition, long-term accelerated molecular evolution, and reduced genome size; these trajectories in certain gut associates are similar to, albeit less extreme than, patterns observed in many obligate intracellular symbionts of insects [12–15].

In light of the patterns above, gut-associated bacteria of insects represent intriguing candidates to explore genomic consequences of symbiotic transitions. Insect gut microbiota are less diverse than those found in vertebrate species with adaptive immune systems [2, 13, 16–19]. Within certain insect hosts, both individual bacterial taxa and whole communities have been reported to maintain a high level of host fidelity, resulting in relationships of remarkable stability over evolutionary timescales [11, 12, 15, 16, 20–22]. Stable associations between insect hosts and extracellular gut associates have been described across several host orders and may offer valuable insights into genomic consequences of inhabiting the gut niche [14].

These genomic consequences are profoundly influenced by transmission mode of a given symbiosis. Broadly, extracellular gut associates of insects fall into three distinct transmission modes, which is a major determinant shaping symbiont genome evolution. These include environmental acquisition, social transmission, or specialized maternal transmission. Examples of each transmission type, respectively, include environmentally acquired *Burkholderia* symbionts of bean bugs that localize to cavities along the midgut (environmental acquisition) [23]; hindgut microbiota of bumblebees transmitted via trophallaxis and coprophagy (social transmission) [24]; midgut associates of stinkbugs transmitted via egg-smearing, symbiont capsules, or nutrient-rich jelly secretions (specialized maternal transmission) [12, 15].

Transmission mode may impact symbiont genome evolution for two main reasons. First, while all gut associates likely have some requirement to survive outside of their host niche [14], the portion of the lifecycle spent outside of hosts may vary with transmission mode. For instance, environmentally acquired symbionts may spend a considerable portion of their lifecycle outside of hosts; by contrast, strictly maternally transmitted microbiota are expected to spend nearly all of their lifecycle associated with hosts and thus become more specialized to the host niche, potentially leading to gene loss. Socially transmitted microbiota may comprise an intermediate point along this spectrum, showing moderate levels of specialization to the host niche, though tempered by interactions with other bacteria or infrequent host switching.

Second, distinct transmission modes are expected to generate varying levels of host-symbiont stability. Environmentally acquired microbiota are expected to show least stability, maternally transmitted microbiota the most, and socially transmitted microbiota an intermediate level. In agreement with this prediction, current studies suggest that host-symbiont phylogenetic congruence, while strongest under strict maternal transmission, is also significant under social transmission, suggesting that sociality may promote vertical transmission [14, 21]. In this sense, socially transmitted microbiota may represent a transition between free living existence and a stably inherited, host-reliant symbiotic lifestyle.

Recent studies of gut microbiota suggest that distinct transmission modes indeed affect trajectories of symbiotic evolution. Genome evolution under the most extreme symbiotic lifestyle of strict maternal transmission and obligate intracellularity provides a useful point of reference. It is well known that hallmarks of long-term intracellularity include severe genome reduction, accelerated substitution rates, and (often) AT biased nucleotide composition [13]; these traits are likely explained by a combination of relaxed selective constraint, accelerated mutation rates, and strong genetic drift due to transmission-related bottlenecks in obligate endosymbionts [25]. Recent studies have demonstrated that extracellular, strictly maternally transmitted stinkbug symbionts show strikingly similar patterns of reductive genome evolution, suggesting that transmission mode -- rather than an intracellular existence *per se* -- is a major contributing factor [12, 15]. Such extreme reductive genome evolution may be constrained in most extracellular gut microbiota (albeit to varying degrees), due to metabolic requirements of free living survival and occasional host-switching [14]. In agreement with that prediction, recent genomic studies have found that some gut microbiota show modest trends toward AT bias and genome reduction, especially among socially transmitted gut associates [14]. Additional studies of gut microbiota across transmission modes are needed in order to understand how transmission affects bacterial lifecycles and resulting consequences on symbiont genome evolution.

In this study, we survey the bacterial communities associated with an abundant eusocial insect, the red carpenter ant, *Camponotus chromaiodes.* We characterize a novel group of Acetobacteraceae, or acetic acid bacteria (AAB), that dominate the *C. chromaiodes* gut community. Further, we demonstrate that these AAB occur in some additional *Camponotus* species screened here, and group phylogenetically with AABs detected in prior studies of other ants. The ant-AAB association represents a useful model for assessing the evolutionary consequences of persistent host association on gut microbes. AAB are known to colonize gut-associated niches across numerous insect species [7, 26, 27], putatively aiding in a range of faculties from metabolite digestion to protection from pathogens. AAB often thrive in the slightly acidic (pH range of 4.5–6.5), micro-oxic gut environment, where they establish tight associations with the gut epithelium, provision various polysaccharides contributing to biofilm formation, and help to structure the gut community by decreasing pH and excluding pathogens [7].

Through a broader phylogenetic analysis of these *Camponotus* AAB and other AAB lineages, we present the first evidence of a novel, monophyletic, and deeply divergent clade of ant-associated gut microbiota positioned in the Acetobacteraceae. We found that the 16S rDNA gene of these ant-associated AAB shows accelerated nucleotide substitution rates, elevated AT content, and decreased predicted stability of the rRNA secondary structure. These features resemble patterns found in coevolved, socially-transmitted gut associates of bumblebees [28]. Such patterns have not been found in other well studied ant associates, such as *Opitutales* taxa often associated with *Cephalotes* hosts [22, 29, 30], as we demonstrate here. These results suggest that AAB may form a persistent association with ants, with significant impacts on the evolutionary trajectory of these bacteria.

## Results

### Two Acetobacteraceae (AAB) OTUs are abundant in the gaster microbiota of *C. chromaiodes*

To characterize the microbiota of *C. chromaiodes*, sequencing of the V4 and V5 regions of 16S rDNA was performed using the Ion PGM instrument. We estimated the empirical error rate of PGM amplicon sequencing by including *E. coli* DH10B in library construction and Ion PGM pyrosequencing. Results supporting a low empirical error rate for length-filtered data are provided in Additional file 1 and Additional file 2.

This amplicon sequencing provided a snapshot of bacterial groups associated with *C. chromaiodes*. Ant samples included three replicates of 3–5 pooled minor workers, from each of six ant colonies (Additional file 3). For four of these colonies, we included the colony queen. Across the ant samples, pyrosequencing generated 343,834 total reads. These datasets were dominated by the intracellular bacteria *Blochmannia* and *Wolbachia,* both known to associate with *Camponotus* [31, 32]. In a previous analysis of 16S rDNA amplicons, *Blochmannia* typically constituted 95-98 % of reads, far outnumbering even *Wolbachia* (unpublished data). In this study, an initial step of digesting amplicons with a *Blochmannia*-specific restriction enzyme (PacI) reduced the abundance of *Blochmannia* reads substantially, to 7.5 % (25,836/343,834 reads). We suspect these remaining *Blochmannia* sequences were due to incomplete PacI digestion of *Blochmannia* 16S rDNA*.* Since the *Wolbachia* 16S rDNA sequence does not contain a PacI restriction site, it was not depleted by the digestion and remains the dominant OTU, or 92 % (316,371/343,834 reads). Neither of the intracellular symbionts are thought to be members of the extracellular gut microbiota: *Wolbachia* display weak tropism for the gut but do not inhabit the gut lumen [33, 34]; *Blochmannia* are housed in specialized host cells intercalated among the gut epithelium and in ovaries [35].

Because the vast majority of sequence reads matched the endosymbionts *Blochmannia* or *Wolbachia*, our sampling of other bacterial groups had low coverage: 1,627 non-endosymbiont reads across all ant samples, or 1,557 reads after removing OTUs with <1 % frequency in the total ant dataset (median of 27 non-endosymbiont reads per sample; Table 1). Despite this low coverage, several striking patterns emerged when analyzing the non-endosymbiont OTUs (Table 1). At the level of ant colony, Proteobacteria and Actinobacteria were the only stable phyla, consistently present across one or more samples per colony. Accounting for most of the widespread distribution of Actinobacteria was a *Nocardia sp.* distributed across 35 % of minor workers and all four queen samples; the total representation of *Nocardia* amounted to 4.6 % of non-endosymbiont reads. Within the Proteobacteria, the class Alphaproteobacteria was dominant in most samples, comprising 75–100 % of Proteobacteria in nearly all samples (Table 1). This trend is largely explained by the high abundance of two distinct OTUs in the family Acetobacteraceae. At least one of these OTUs was detected in 20/22 (91 %) of samples. They dominate the non-endosymbiont OTUs detected, comprising 79.8 % of that bacterial community (AAB1: 52.8 %, AAB2: 27 %; Table 1).

**Table 1:**
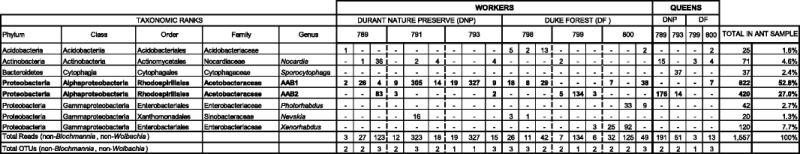
Abundance (read count) of Bacterial OTUs detected in *Camponotus chromaiodes*. Read counts are based on 16S amplicon sequencing. “-” indicates zero reads detected. Endosymbiont reads (*Blochmannia* and *Wolbachia*) have been removed from the read counts shown. Only OTUs comprising >1 % of total reads are shown. Each worker sample represents a pool of 3–5 surface-sterilized whole gasters. Colonies are demarcated by a dashed line line and listed with a three digit identifier. The total % contribution of each OTU (right-most column) reflects the total read count of that OTU, among the 1,557 non-*Blochmannia*, non-*Wolbachia* reads for all 22 ant samples, after excluding OTUs <1 %. AAB1 & 2 are displayed in boldface

### AAB are localized to the *Camponotus* gut tract

Because we chose to use whole gasters (rather than dissected gut tracts) to characterize the gut microbiota via pyrosequencing, we assessed the efficacy of our sterilization protocol (#1; see Methods) and another standard protocol for removing exogenous bacterial cells or DNA that could adhere to the cuticle (#2). We also assessed the relative bacterial load expected for unsterilized specimens. After extracting gDNA from dissected tergite pools (portions of the gaster cuticle), we screened for the presence of bacterial 16S rDNA via PCR with universal bacterial primers (9 F and 1046R) [36]. Neither sterilization approach (#1 or #2) yielded detectable amounts of gDNA via absorbance measurement at 260 nm, though both samples yielded positive, albeit very weak, PCR amplification. Conversely, unsterilized tergite segments yielded detectable levels of gDNA for both assays; detectable gDNA via absorbance at 260 nm suggests much higher amounts of bacterial DNA in the unsterilized samples, suggesting that both sterilization techniques were effective in removing bacterial DNA. However, positive amplification of 16S rDNA from sterilized tergite samples suggests that the source of bacterial DNA was due to incomplete removal of bacterial cells or DNA during sterilization, or an artifact acquired during DNA extraction, as extraction kits are not sterile [37].

In order to determine the host tissue in which AAB reside, we performed PCR on various samples with AAB-specific primers. We found that the tergite pools described above, regardless of sterility, did not yield detectable amplification of either AAB1 or AAB2. This rules out the possibility that AAB1 and AAB2 detected here reflect bacterial DNA on the cuticle or in the DNA extraction kit (as the kit was used to prepare tergite DNA). Conversely, AAB1 and AAB2 were successfully amplified from dissected whole gut tracts (fore-, mid-, and hindgut combined) from individual worker ants, collected the same day and surface sterilized following approach #2. Sequences of resulting PCR products were identical to sequences detected through pyrosequencing. Thus, in our description of the *C. chromaiodes* microbiota below, we are confident that AAB1 and AAB2 can occur in the host gut. However, the other OTUs detected through amplicon pyrosequencing (Table 1) could conceivably occur in the gut, elsewhere in the gaster, or on the cuticle. Three lines of evidence argue against these OTUs being contaminants of the DNA extraction kit: (i) their presence varied across biological samples (all of which were extracted with the same kit; (ii) nearly all OTUs belong to genera that are not among the bacterial groups that are known kit contaminants [37]; (iii) related OTUs in many samples have been detected in the gut of other ant species (see Discussion).

### Strain-level variation occurs within AAB OTUs, and screens across host species and geographic regions

We analyzed the genetic diversity within each AAB OTU at the V4-V5 regions from data generated by pyrosequencing. Within the AAB2 OTU, we detected two nucleotide polymorphisms in the V4-V5 regions. Each had an overall frequency of 3.95 % in the read dataset, far above for the frequency expected for sequencing errors (average substitution error frequency of 0.34 %; Additional file 2). Due to the relatively short length of the V4-V5 amplicon (~400 bp), we did not include the AAB2 variant in phylogenetic analyses of near full-length sequences.

To assess the prevalence of the ant-AAB relationship, we screened additional *Camponotus* species from distinct geographic locations with AAB-specific PCR, in some cases confirming specificity via Sanger sequencing of resulting PCR products. These screens demonstrated that AAB1 was present in *C. pennsylvanicus* and *C. castaneus* from the northeastern US (Massachusetts) and *C. castaneus* from North Carolina (Additional file 4). AAB2 was found only in *C. chromaiodes* from colonies that originated in North Carolina (Additional file 4).

**Fig. 1:**
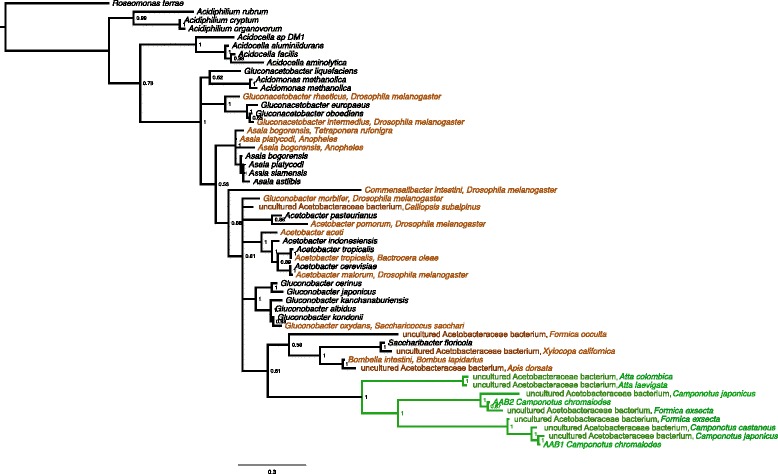
Bayesian phylogeny of Acetobacteraceae 16S rDNA. Phylogeny is based on a filtered alignment of 1,248 nucleotide positions. Bayesian inference was based on the GTR + Γ + PInv model of nucleotide substitution and analyses run for 10,000,000 generations. Values at nodes reflect posterior probabilities after a 25 % burn-in. Taxa are colored based on lifestyle and host group: monophyletic ant-specific AAB clade (*green*); gut associates of various insects (*orange*); free living taxa (*black*). The host name is listed after the bacterial taxon name. 16S rDNA sequences of AAB1 from *C. chromaiodes* and *C. castaneus* (included in the tree shown) differ by two nucleotides. Sequences of AAB1 from *C. chromaiodes* and *C. pennsylvanicus* were identical over the region sequenced (*E. coli* coordinates 828–1046). *Roseomonas terrae* is the outgroup

**Fig. 2:**
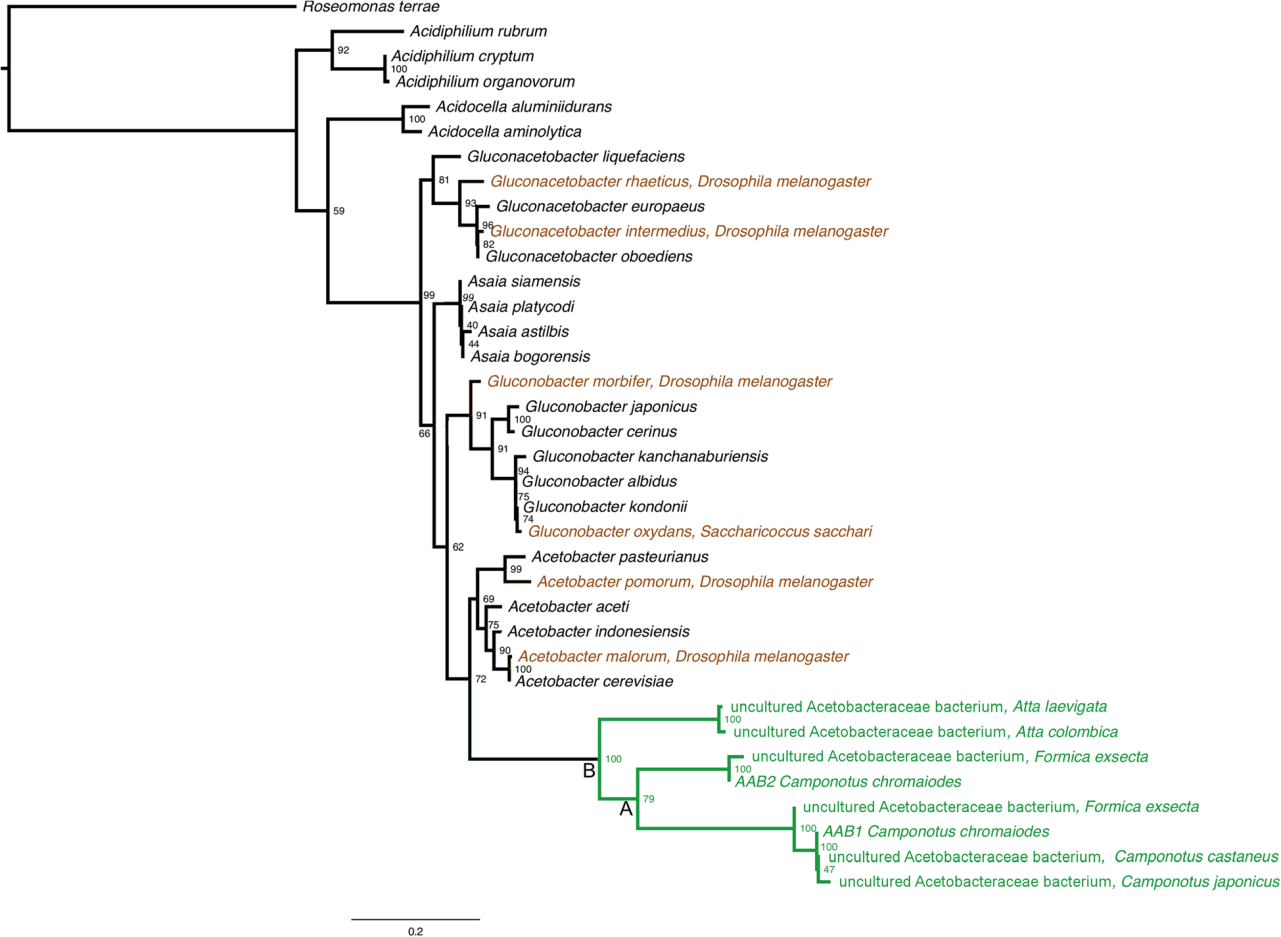
Maximum likelihood phylogeny of Acetobacteraceae 16S rDNA. Alignment of 1,333 unambiguous nucleotide positions was performed with SSU-align. The tree was reconstructed using a maximum likelihood approximation with a GTR + Γ + PInv model of nucleotide substitution. Node support was generated from 1,000 bootstrap resamplings. Taxa are colored based on lifestyle and host group: monophyletic ant-specific AAB clade (*green*); gut symbionts of various insects (*orange*); free living taxa (*black*). *Roseomonas terrae* is the outgroup. Letters at select nodes correspond to nodes used for relative rate analysis, as listed in Table 2

For select samples in this cross-species screen, we amplified and (Sanger) sequenced a longer region of the 16S rDNA gene, using a combination of OTU-specific and general bacterial primers. AAB1 from one isolate of *Camponotus castaneus* (854.4; Additional file 4) showed two polymorphisms in the V2 region, when compared to the AAB1 sequence from *C. chromaiodes*. One polymorphism reflected a clear nucleotide transition, and the second reflected a mixed base signal, indicating a G/A sequence polymorphism within the *C. castaneus* individual sampled*.* At this position, chromatograms from both sequencing orientations showed a greater fluorescent intensity for G, as compared to A (the consensus base in the *C. chromaiodes* sequence). This AAB1 variant from *C. castaneus* is included in the phylogenies presented (Figs. 1 and 2).

### *Camponotus*-associated AABs join other ant associates in a deep, well-supported clade

Phylogenetic analyses of 16S rDNA sequences showed that AAB1 and AAB2 group together, along with other Acetobacteraceae OTUs sampled from ant hosts or ant nests. This group of ant-associated Acetobacteraceae form a deeply divergent, monophyletic clade that is well supported based on Bayesian analysis (1.0 posterior probability; Fig. 1) and maximum likelihood analysis (100 % bootstrap support, Additional file 5).

Based on current sequence data available from the Ribosomal Database Project (RDP) [38], this clade apparently lacks any non-ant associates. That is, among the RDP 16S rRNA database (>3 million sequences), including environmental isolates and plant and animal associates, we did not detect any non-ant associated sequences that were putative congeneric bacteria (>95 % similar) with the ant AAB. By comparing near full length 16S rDNA sequences of ant-associated AAB to the entire RDP database, we found that *Camponotus* gut isolates were the only sequences with an aligned similarity score greater than 92.6 % to AAB1, or greater than 94.4 % for AAB1. For the Attine AAB lineage, the closest non-ant relative had an aligned similarity score of 92 %. To determine if any sequences in RDP would conflict with the monophyly of the ant-associated AAB clade, we chose 60 sequences that showed the highest sequence similarity to AAB1, AAB2, and/or the Attine-associated AAB via SeqMatch, as well as select sequences that we found to be close relatives in the Bayesian and ML analyses here. Within RDP, we estimated a distance-based tree of aligned sequences. Only ant-associated bacteria grouped with each of the three sequences above. The RDP tree supported the grouping of AAB1 and AAB2 together. While the analysis could not resolve the position of Attine AAB, the RDP results do not conflict with the monophyly of the three lineages demonstrated by the more extensive Bayesian and ML analyses in this study.

As expected of a single-gene phylogeny, some relationships in the Bayesian and ML analyses were not well resolved. For instance, while the ant-associated AAB clade was extremely well supported, the identity of its sister group was not. Albeit with weak support (posterior probability of 0.61), Bayesian analysis suggests the sister group includes various bee associates [39] (Fig. 1). ML analysis was unable to resolve this and other relationships (Additional file 5).

To ensure that we could accurately assess relative substitution rates (below), we used an alignment of fewer taxa to generate a robust maximum likelihood tree, as a guide for substitution rate analysis (Fig. 2). Phylogenetic reconstruction was based on an alignment of 1,333 unambiguous nucleotide positions of 16S rDNA. Most nodes were well supported, and all relationships were identical between maximum likelihood (Fig. 2) and Bayesian (Additional file 6) approaches.

### The ant-associated AAB clade evolves several-times faster than other Acetobacteraceae

We compared distinct molecular clock scenarios to determine the likelihood of distinct rates across this phylogeny (Table 2). Allowing a distinct rate for the ant-associated AAB clade (Clade B in Fig. 2, Table 2) resulted in a significant improvement in likelihood score, when compared the null hypothesis of a global clock (uniform rate) (*p* < 10^−30^). The same analysis also estimated a 7.6-fold rate increase in Clade B, compared to the single rate inferred for all other lineages. This clear result argues for a statistically significant, several-fold rate increase in the ant-associated AAB clade, compared to a single rate calculated for all other lineages.

**Table 2:** Comparison of molecular clock models and relative substitution rates of 16S rDNA. All likelihood values were calculated using PAML4. The tree shown in Fig. 2 was used to calculate relative rates and the likelihood scores for the global clock model and both local clock models. The null hypothesis of each comparison was a global clock (uniform rates) model. Clade IDs correspond to those listed in Fig. 2. All rates are relative to the rest of the tree, without the inclusion of the outgroup taxon, *Roseomonas terrae*. Abbreviations: DF, degrees of freedom; LR, likelihood ratio

Comparison	DF	Number of distinct rates	Clade ID(s) with distinct rate	LR	Null hypothesis (−lnL0)	Alternative hypothesis (−lnLA)	p	Relative substitution rates
Global clock vs. local clock	36	2	B	227.91	6479.11	6365.16	9.84E-30	7.6
Global clock vs. local clock	37	3	A, B	230.56	6479.11	6363.83	8.12E-30	8.9, 6.1

To explore rate variation within the ant-associated AAB clade, we compared an additional multi-rate model to the null of a global clock. In this case, the local clock model defined three distinct rates: a distinct rate for both of Clades A and the remainder of B (i.e., all lineages within Clade B that are not part of subclade A), and a third rate for all other lineages (Fig. 2, Table 2). This three-rate model was also significantly better than the uniform rate, and supports rate acceleration throughout the ant-associated clade. Specifically, under this model, Clade A was found to have a 8.9-fold rate increase, and (the remainder of) Clade B a 6.1-fold increase. Despite increased resolution, this model is only marginally more likely than the simpler, two-rate model. Because within-clade rate heterogeneity can bias inferences of local rate estimation [40], we cannot test whether it is significantly better than the simpler two-rate model.

### *Camponotus* AABs show increased AT content and reduced RNA stability, comparable to known socially transmitted bacteria

**Fig. 3:**
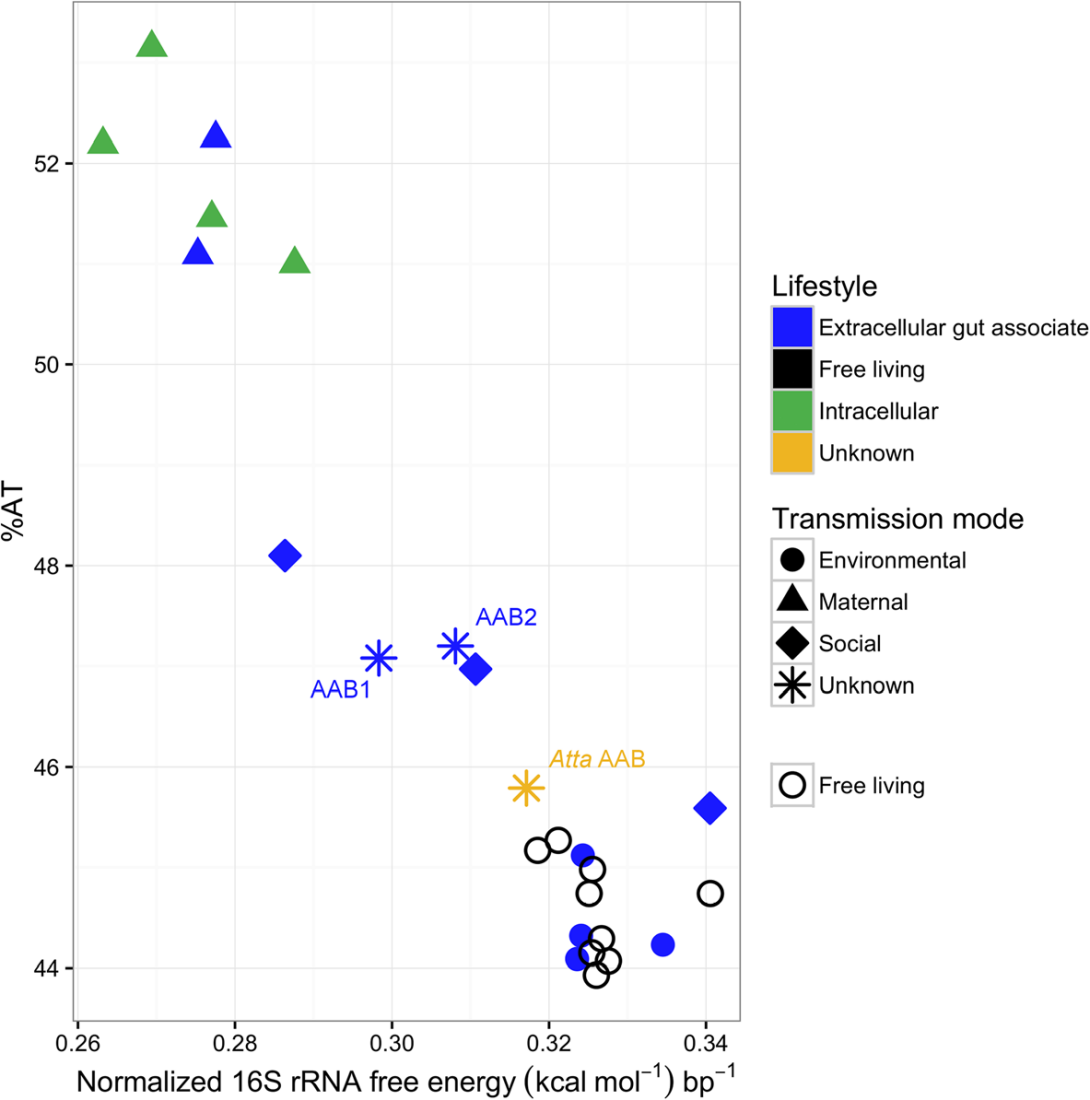
Relationship between AT content and normalized predicted free energy of 16S rRNA, showing the grouping of sequences by bacterial transmission mode. Free energy estimation was constrained to known secondary structure interactions (in the *E. coli* rRNA molecule) and calculated using the Turner 2004 model. Analysis was based on the region corresponding to *E. coli* coordinates 16–1477. Shape indicates transmission mode (when known), and colors indicate bacterial lifestyle. AAB1 and AAB2 from *C. chromaiodes* are marked (blue stars; extracellular gut associates of unknown transmission mode). The closely related AAB lineage from *Atta laevigata* appears as a yellow star (unknown lifestyle and unknown transmission mode). The names and values for all points are provided in Additional file 7. Lifestyle designations are with respect to insect hosts

Normalized estimates of thermodynamic stability (Fig. 3; Additional file 7) suggested that 16S ribosomal rRNA of both AAB OTUs were less stable than all free living bacteria analyzed. The predicted stability of AAB1 and AAB2 is comparable to extracellular gut associates of other social insects, such as *Snodgrassella alvi* and *Gilliamella apicola* of bees (Fig. 3; Additional file 7). These and other extracellular gut associates had higher stability than the intracellular or strictly maternally transmitted bacteria analyzed here. These values are based on the region of 16S rDNA corresponding to *E. coli* coordinates 16–1477 and were constrained to known secondary structure interactions of 16S rRNA molecules. Notably, predicted stability of 16S rRNA molecules appears to be negatively correlated with AT content (Fig. 3).

## Discussion

Several lines of evidence support the hypothesis that Acetobacteraceae is a frequent gut associate in *Camponotus* species and perhaps other ant groups. First, 16S rDNA amplicon sequencing of local *C. chromaiodes* microbiota showed that at least one of two Acetobacteraceae OTUs (AAB1 or AAB2) occurred in most host samples analyzed and was present in both workers and mated queens. Second, although some of our samples were based on whole ant gasters, we confirmed through analysis of dissected samples that AAB1 and AAB2 indeed occur in the gut tract of *C. chromaiodes.* Third, using AAB-specific PCR primers, we detected AAB1 in additional *Camponotus* species and regions: *C. castaneus* from North Carolina, and *C. castaneus* and *C. pennsylvanicus* from Massachusetts. Fourth, our phylogenetic analysis documented that AAB1 and AAB2 belong to a deeply diverging, monophyletic group that includes associates of diverse ant species (detailed below) and, based on current data, is restricted to ant associates (Fig. 1; Clade B in Fig. 2). This phylogenetic pattern suggests that the clade may be specialized for association with ants. Fifth, at the 16S rDNA gene, molecular evolution of the ant-associated AAB clade exhibit trends that resemble stably inherited symbionts: significantly accelerated rates of evolution, AT-biased sequence composition, and (perhaps a result of AT bias) destabilization of predicted rRNA stability. Combined, these data suggest the association of this AAB clade with ants may be specialized and quite old.

### Monophyly of ant-associated AAB

In more depth, the ant-associated AAB clade contains two lineages that include OTUs (AAB1 and AAB2) that we amplified from *C. chromaiodes* and (in the case of AAB1) from *C. castaneus* and *C. pennsylvanicus* samples. The detection of these two lineages in association with ants is not new. Previous studies have identified ant associates that are closely related to AAB1, including a gut associate of *C. japonicus* [41] and a bacterial associate of *Formica exsecta* detected in a transcriptome analysis [42]. Previous work has also detected bacteria that are closely related to AAB2, including a dominant taxon in the crop of lab-reared *Camponotus fragilis* [43], a gut associate of *C. japonicus* [GenBank: KM974908.1], and another *F. exsecta* associate [42]. In addition, unpublished sequences from gut tissue of adult *Cataglyphis bicolor* [GenBank: KF419351] and *Cataglyphis fortis* [GenBank: KF419347] group robustly (posterior probability =1.0, unpublished data) with AAB2. These two sequences were not included in the final trees presented here, due to their relatively short sequence lengths.  In sum, while several studies have documented AABs in association with ants, often verifying their location in the gut tract, to our knowledge this is the first demonstration that various ant-associated AABs form a monophyletic group and may represent a specific association with ants. In light of these diverse associations with various ant groups, and the known role of Acetobacteraceae as gut symbionts in other insects [7, 27, 44], we propose that these two AAB lineages may be beneficial gut-associated symbionts of *Camponotus*, and potentially of the subfamily Formicinae more generally.

Within the ant-specific AAB clade, a third, basal lineage consists of bacteria associated with Attine ants. These samples include bacteria found within microbial assemblages of refuse dumps of Panamanian attine ants [45], and a related OTU from an unidentified source of Brazilian *Atta laevigata* [GenBank: KF248847]. In addition, this group includes an associate of the ant *Megalomyrmex staudingeri*, a social parasite of several Attine genera [46] (posterior probability = 1.0, unpublished data). (Sequence was not included in the trees presented, due to relatively short length.) While further sampling is needed, the intriguing position of this third group as a basal lineage to known Formicinae associates above (AAB1, AAB2, and relatives) raises the possibility that an association between this clade and ants is ancient.

Detection of a related AAB in attine refuse dumps is consistent with this bacteria being part of the gut microbiota, as dumps contain the bodies of dead workers [47] and are likely to reflect a mixed composition of microbial communities from various sources. While the context of the *Atta-*AAB association is unclear at this time, our combined analyses of AAB1 and AAB2 are consistent with trends observed in persistent gut associates rather than associates that are incidentally acquired from the environment, e.g., via forage fed upon by ants. Moreover, AAB1 or AAB2 have not been detected in previous screens of ant gardens or soil chambers [48]. Further arguing against incidental environmental acquisition, we found that among numerous 16S rRNA sequences available in the RDP database, including those most similar to the ant-associated AAB, no non-ant associated bacteria disrupt the monophyly of the clade described above. If members of this AAB group exist among environmental isolates, in association with non-ant animals, or in association with plants, they have not yet been described to our knowledge.

As expected of a single-gene phylogeny, many relationships in the Acetobacteraceae tree were difficult to resolve, including the sister lineage to the ant-specific clade. Bayesian inference supports (albeit weakly) that the sister lineage includes Acetobacteraceae gut symbionts of bees [39, 44], as well as an associate of the ant *Formica occulta*. Certainly, further phylogenetic analyses that include additional genes are needed in order to better resolve this relationship. If evidence for a close relationship between bee and ant associates is verified, it is possible that this bacterial lineage shares pre-adaptive traits fostering colonization of hymenopteran guts [9]. Interestingly, associations between Acetobacteraceae and ants apparently have occurred multiple times independently. That is, the ant-specific AAB clade detailed above does not contain all AAB detected in ants. A comprehensive survey of ant microbiota [49] identified *Asaia bogorensis* and an unspecified OTU that were associated with *Tetraponera* and *Formica* ants, respectively (Fig. 1). Whether these associates represent transient interactions or stable interactions is unclear.

Phylogenetic independence among bacterial associations is not limited to ants. Consistent interactions between *Drosophila* and phylogenetically distinct gut bacteria within the Acetobacteraceae (*Acetobacter pomorum and Gluconobacter morbifer*) [50] have been implicated in specific interactions with host health and disease states [27]. Interestingly, both of these associates occur on relatively long branches, but show a predicted rRNA stability comparable to that of *E. coli,* and the rates of molecular evolution at 16S rDNA is similar to that of free living bacteria (Fig. 3, Additional file 7). In the case of these and other taxa on long branches, additional sampling of related OTUs will help to elucidate whether the long branches are due to shifts in patterns of molecular evolution or an artifact of incomplete sampling.

### Rapid evolution and destabilization at 16S rDNA

In light of the above evidence that the ant-specific AAB clade may represent a long-term association with ants, we tested whether patterns of molecular evolution at the 16S rDNA gene resemble those found in stably inherited bacterial symbionts. We found that patterns of sequence evolution in this group resemble known socially or maternally transmitted gut associates, moreso than environmentally acquired associates or free living bacteria. These patterns include accelerated rates of substitution, AT bias, and reduced stability of 16S rRNA secondary structure, detailed below. The magnitude of rate acceleration surprisingly resembled those detected for some intracellular symbionts.

Significant acceleration in rates of sequence evolution is well established among the most extremely stable symbionts: long-term, maternally transmitted, intracellular mutualists of insects [13]. Numerical estimates of the extent of this acceleration may depend on the gene analyzed and taxa compared, but studies consistently support several-fold rate increases. For example, estimates of the absolute fold-change in substitution rate at 16S rDNA include a ~1.5 to 2.6-fold acceleration in *Buchnera* [51], and up to a 15 to 29-fold acceleration in *Blochmannia* [52]. Likewise, a significant, ~6.5-fold rate acceleration was detected in gut bacteria of stinkbugs, an extracellular associate that shows extremely high fidelity of maternal transmission and patterns of reductive genome evolution that resemble those of long-term intracellular associates [12]. This observed rate acceleration may reflect a combination of processes that differentially affect stably transmitted symbionts, such as relaxed selection, increased mutation rate (e.g., if DNA repair genes are lost), and reduced efficacy of purifying selection due to a reduction in N_e_. These evolutionary mechanisms have been distinguished in some symbiont groups through analyses of several genes [25]; however, they are impossible to distinguish based on 16S rDNA data alone.

Our results indicate that the clade containing AAB1 and AAB2 may experience an acceleration of sequence evolution that is comparable to the examples above. At 16S rDNA, the ant-specific clade is evolving at a significantly faster rate than other Acetobacteraceae included in the analysis. That is, a model allowing a faster rate for this clade represents a significant improvement over the null model of uniform rates across the tree. While the fold-increase in rate depends on the taxa included, we estimate that 16S rDNA gene is experiencing ~7.6-fold acceleration in substitution rates, as compared to free living and other insect-associated AAB included (Fig. 2; Table 2).

In addition, AT bias characterizes most long-term intracellular associates, and more recently has been documented in extracellular gut associates with maternal and social transmission [12, 53]. Like rate acceleration and often coupled with it, increased AT bias may reflect a combination of increased mutational pressure, or a reduction in the strength or efficacy of purifying selection against GC → AT changes. We detected atypically high AT content of 16S rRNA for AAB1 and AAB2 (~47 % AT), compared to other host-associated and environmental bacteria (Fig. 3; Additional file 7).

Similar to reports on obligate endosymbionts of insects [54], we found a notable decrease in 16S rRNA stability for AAB1 and AAB2 (Fig. 3). Normalized folding free energy of 16S rRNA (spanning *E. coli* coordinates 14–1445) is ~7–8 % lower in both AAB OTUs than free living AAB. By contrast, across the same region, various species of the obligate endosymbiont, *Blochmannia*, are ~10–11 % less stable than *E. coli.* Unlike previous studies [54], we did not distinguish whether base substitutions are phylogenetically independent; nor can we rule out the possibility that the reduced stability simply reflects the observed increase in AT content of this molecule. Interestingly, the stability difference between AAB1 and AAB2 is large (~3.2 % less stable in AAB1), despite a modest shift in AT content (0.1 %). This shift in stability across similar AT content suggests factors other than base composition contribute to destabilization. Comparative genomic data could shed light on the mechanisms driving the overall destabilization of AAB1 and AAB2, and the notable difference between them. The AT content of 16S rDNA from Attine-associated AAB was slightly elevated, but its structural stability was comparable to free living bacteria or environmentally acquired symbionts.

Although evidence based on one gene is limited, this combined pattern of rate acceleration, increased AT content, and reduced rRNA stability in ant-specific AAB1 and AAB2 suggests that their molecular evolution may resemble known socially and maternally transmitted symbionts. A similar, though more extreme trajectory typifies symbionts with strict maternal transmission, including most obligate endosymbionts. These parallels suggest that socially transmitted bacteria may experience some of the evolutionary forces that shape long-term endosymbionts, albeit to a lesser degree. Such forces may include intensified mutation rates via relaxed selection (though constrained by the requirement for survival outside of hosts [14]) and possibly fixation of deleterious changes by genetic drift (though mitigated by host-switching and potential recombination with other members of the microbiota [11]). Further studies will shed light on the extent to which transmission mode contributes to this evolutionary trajectory and the underlying mechanisms involved.

Within each AAB OTU, we detected polymorphisms at 16S rDNA. Variation within a gut associated OTU has been demonstrated in extracellular gut associates of bees [55], and may predict strain-level variation in gene content and ecological role. Specifically, within AAB2, we detected two polymorphic alleles in the V4-V5 region. Likewise, within AAB1, we detected polymorphisms across host species, although our limited sample does not allow us to assess host specificity of these variants. Further studies are needed to explore whether this strain-level variation arises from niche specialization or reflects neutral changes, e.g., under an elevated substitution rate.

### Potential transmission routes and caste variation

Although clarifying the transmission routes of AAB1 and AAB2 will require experimental data, the patterns above suggest the possibility of social transmission via nutritional or other interactions among ant nestmates. As a eusocial host, intimate contact within colonies creates opportunities for transfer of gut microbiota among kin (e.g., between queen and her offspring, and among workers) via trophallaxis. Such nutritional exchanges may transfer gut microbes and promote stability of the gut community [21, 56, 57]. Anal trophyllaxis (i.e. feeding on anal secretions) likely fosters microbiota transmission in the few ant groups that perform this behavior (e.g., *Cephalotes*) [22, 49] and may contribute to the phylogenetic stability of the microbiota in *Cephalotes* [22]. In most ants, including the tribe Camponotini, trophallaxis is mouth-to-mouth (stomodeal trophyllaxis), involving the regurgitation of crop contents. This social feeding transmits important nutritional resources, and might also transmit key gut microbes.

On a community scale, the microbiota of *C. chromaiodes* had low taxonomic diversity, and many taxa occurred sporadically. However, some taxa were identified in several samples, and relatives have been documented in additional ant species. The two AABs were, by far, the most abundant within most samples and widespread across samples. In addition, a *Nocardia sp.* was detected repeatedly, within and across host colonies*.* Interestingly, two OTUs with 100 and 98 % sequence identity to the *Nocardia* sp. have also been detected in the guts of *C. japonicus* [58] and the camponotine *Polyrhachis robsoni* [59], respectively, though the broader prevalence of this relationship is unknown.

Microbial communities from colony queens were similar to those of workers. With one exception (queen from colony 799; Table 1), queens possessed one AAB OTU or the other, but not both. However, the AAB OTU detected in the colony queen was not necessarily the numerically dominant AAB in workers from the same colony. The *Nocardia* OTU described above was also consistently recovered across colony queens. The processes generating such differences between queen and worker microbiota are uncertain, but may include transfer of gut bacteria among workers from different nests, distinct ecological roles of queens and workers within the colony, and shifts in the gut community as queens age. The colonies sampled were large, well-established nests, meaning that resident queens could be several years old.

Exploration of transmission routes and caste variation will benefit from a deeper sampling of the gut community. While our efforts to deplete the abundance of 16S rDNA amplicons from *Blochmannia* were successful, *Wolbachia* contributed significantly to our sequencing effort. In the context of gut community surveys, the alimentary tract is relatively large among the Formicidae, and likely amenable to microdissection of luminal contents for targeted characterization of those communities, as has been performed in termites a [2]. Combining microdissection with high-throughput sequencing [60] will facilitate deeper exploration of gut communities among insects that also house abundant endosymbionts near the gut.

In sum, insect hosts continue to offer fertile ground to explore evolutionary processes shaping host-microbe interactions, including gut-associated symbioses. Exploration of diverse host systems will help to uncover fundamental principles shaping these relationships, including the influence of transmission mode on symbiont evolution. While our results point to distinct patterns of sequence evolution in the ant-specific Acetobacteraceae clade, additional studies are needed to clarify the evolutionary mechanisms driving these observed patterns and to explore potential genome-wide impacts. In addition, experimental work is required to test the hypothesis of social transmission and to understand the potential functions and host fitness effects of this bacterial group.

## Conclusions

In concert, the data presented here suggest that Acetobacteraceae symbionts represent abundant and dominant members of the gut community of *Camponotus chromaiodes.* The two most abundant members, AAB1 and AAB2, belong to a novel and deeply divergent, monophyletic clade of ant-associated bacteria. Relatives of these taxa were recovered from two additional *Camponotus* species, and are known from prior studies to occur in additional ant groups. Members of this ant-specific AAB clade display patterns of molecular evolution at the 16S rDNA gene that resemble patterns found in socially transmitted gut symbionts of bees and, in a more extreme form, cospeciating, maternally transmitted gut associates of stinkbugs. Specifically, these features include elevated AT content, a ~7.6-fold increase in substitution rates at 16S rDNA, and a notable decrease in the predicted stability of 16S rRNA. Cumulatively, these results are consistent with the hypothesis that this AAB clade may represent a long-term gut associate of ants.

## Methods

### Ant collection

Ants were collected for two purposes: to characterize the microbiota of local *C. chromaiodes* colonies, and to screen *Camponotus* of various species and geographic regions for AABs detected. For the microbiota characterization, we sampled several minor workers and, when available, the mated colony queen from each of six local *C. chromaiodes* colonies. All specimens were immediately placed in 100 % ethanol and transported to Duke University upon collection. Collection information is available in Additional file 3. For the targeted AAB screen across *Camponotus* species and geographic regions, we used ants that were collected from 2008 to 15. This collection information is available in Additional file 4. For all samples, vouchers are available upon request.

### Sample preparation for microbiota characterization: surface sterilization, genomic DNA (gDNA) extraction, and *Blochmannia* depletion

Microbiota characterization of local *C. chromaiodes* was based on surface-sterilized gasters. All samples were surface-sterilized in 100 % ethanol and washed 4–6 times in sterile 50 mL vials filled with DEPC water. Surface sterility was confirmed by negative PCR on the final wash bath. We call this protocol surface sterilization approach #1.

gDNA was extracted from 3 to 5 pooled, surface-sterilized worker gasters per sample, or the single gaster of the colony queen (Additional file 3), using the Qiagen (Hilden, Germany) DNEasy kit according to a standard protocol. We chose to use whole, surface-sterilized gaster samples rather than dissected gut tissue, as dissection may increase contamination. Because surface-sterilized gasters were used, we cannot conclude that all bacterial taxa detected were gut-associated (our later use of dissected gut samples, described below, confirmed that the AAB detected in this characterization resided in the gut).

*Blochmannia*, the long-term intracellular endosymbiont of *Camponotus* and related genera, far outnumbers extracellular gut associates. Thus, in order to deplete *Blochmannia* 16S rDNA abundance, 500 ng of gDNA per sample were treated with NEB (Ipswich, Massachusetts, USA) PacI enzyme according to the manufacturer’s protocol. The *Blochmannia chromaiodes* 16S rDNA gene contains a unique PacI site from coordinates 124–131. The site is rare in other bacteria, occurring in just 0.38 % (sporadically distributed) of sequences in the RDP 16S database. This rarity makes it extremely unlikely that 16S rDNA of other gut-associated taxa would contain this restriction site. To ensure the specificity of our approach, we amplified 16S rDNA with universal Bacterial primers from undigested ant gDNA, digested the resulting PCR product with PacI, and visualized the digested products on an Agilent Bioanalyzer. The products of digestion included two bands corresponding to the expected fragment sizes for *Blochmannia*. We then gel-purified and sequenced the digested amplicon using an ABI 3730xl (Life Technologies, Carlsbad, CA, USA). The sequences of these digested fragments showed clean *Blochmannia* sequences, indicating that 16S rDNA of other bacterial species were not digested by PacI. In order to reduce the contribution of *Blochmannia* to 16S rDNA amplicon pools, PacI-digested gDNA was used as template in the initial PCR for library preparation, below.

### 16S rDNA amplicon pyrosequencing using Ion PGM

#### Selection of region

We targeted the V4-V5 region of 16S rDNA (*E. coli* coordinates 515–926) to maximize the strengths of Ion PGM (Life Technologies, Carlsbad, CA, USA) sequencing. First, the V4-V5 regions are flanked by conserved sequences that have been validated as universal PCR primer sites across Bacteria [61, 62] (5′ primer coordinates 515 and 926; Additional file 8). Second, the length of this region is well suited for the Sequencing 400 kit (410 bp empirical modal read length). Third, by sequencing both the V4 and V5 hypervariable regions, we were able to achieve robust taxonomic resolution at the genus level.

#### Initial PCR reaction

In order to minimize barcode-induced amplification bias during library preparation, we performed a two-step PCR approach [63]. From each PacI-digested gDNA sample, we first amplified a ~918 bp region of 16S rDNA that spans the PacI restriction site in *Blochmannia.* PCR reactions were performed in triplicate and contained 5.05uL nuclease-free water, 1uL 10x High Fidelity buffer (Life Technologies), 0.25uL 10 mM dNTP mix, 0.5uL 50 mM MgCl2, 0.8uL forward primer 9 F, 0.8uL reverse primer mix 926R, 0.1uL Platinum Taq DNA Polymerase High Fidelity (Life Technologies), and 1.5uL of PacI-digested gDNA. Reactions were held at 95 °C for 2 min to denature the DNA, with 25 cycles of amplification proceeding at 95 °C for 20 s, 50 °C for 30 s, and 72 °C for 30 s, we added a final extension phase at 72 °C for 5 min, then cooled at 4 °C. Resulting amplicons were then pooled, cleaned using the Qiagen PCR Purification kit following the manufacturer’s instructions, and digested with PacI following the same protocol as above (for gDNA), to further deplete any undigested 16S copies from *Blochmannia*. All samples were cleaned and run on a 1 % E-gel (Life Technologies) for 13 min. The target (undigested) bands were excised, cleaned, and used as template for Ion PGM libraries below.

#### Ion PGM library construction and sequencing

10-cycle PCRs were set up similarly to above but using barcoded fusion primers to amplify the V4 and V5 hypervariable regions from the 25 cycle amplicons described above*.* All primers used are detailed in Additional file 8. All libraries were quantified using a Qubit 2.0 Fluorometer (Life Technologies), then pooled in equimolar concentrations before being sequenced with an Ion 314 Chip Kit v2, using the Sequencing 400 Kit on the Ion PGM. Sequencing was performed by the Genome Sequencing and Analysis Core Resource at Duke University according to the manufacturer’s protocol for Ion Amplicon Sequencing. This approach generated 658,420 raw reads (across all ant samples and the *E. coli* control library), of which 337,364 passed our quality and length filters and were used in downstream analyses.

### Microbial community analysis

Raw sequencing reads were processed using QIIME 1.8 [64] and UPARSE [65]. All reads were trimmed of sequencing adaptors, barcodes, and forward and reverse primers. Based on our estimates of empirical error rates from an *E. coli* DH10B control library, found that length-based thresholds (universal trimming of reads to 360 bp, and discarding any reads <360 bp) followed by run-specific expected error filtering [65] proved to be the most effective way to reduce sequencing errors (Additional files 1 and 2). Expected error filtering parameters were based upon retaining as many reads as possible while still successfully calling a single OTU for the *E. coli* control dataset. The UPARSE pipeline [65] was used to group operational taxonomic units (OTUs) at a 97 % similarity threshold and to remove chimeric sequences. Taxonomy was assigned to UPARSE-identified OTUs using the RDP Classifier against Greengenes database 13.8 [38, 66]. Relative abundance estimates were generated during data analysis with QIIME.

### Within-OTU polymorphism

To calculate the genetic diversity within an OTU, we used an approach similar to that for error calculation (Additional file 1). As there is no reference sequence for either OTU, we used the representative sequence for that OTU, which was the most abundant sequence. Using the OTU representative sequence as the reference, we aligned all Ion PGM reads to the reference sequence and performed variant calculations as described above. Because indel errors rates can vary widely with pyrosequencing data, we were only able to assess polymorphism frequency for nucleotide substitutions. We characterized a polymorphism as genuine only if its frequency deviated from the empirical substitution error rate by an order of magnitude. Thus, our estimates of OTU heterogeneity are likely conservative.

### Assessment of surface sterilization techniques

To assess the efficacy of the surface sterilization of ant gasters, we sterilized pooled samples of tergites and analyzed samples by PCR to test exogenous bacterial cells or bacterial DNA that could adhere to the cuticle. From each of five minor workers, two to three tergites were dissected under microscope and pooled. We generated three such samples of 13–15 tergites each. Each pool was processed exactly as the experimental samples, but then treated with one of two sterilization regimes. Under sterilization approach #1 (identical to that used above to prepare samples for Ion Torrent sequencing), tergites were vortexed and incubated for ≥5 min in 100 % ethanol, then washed in sterile water until the final wash bath did not yield a positive signal from PCR with universal bacterial primers (as above). Under approach #2, tergites were dipped into 95 % ethanol, soaked for one min in 5 % bleach, and then rinsed in sterilized water*.* We also included a sterilization-free sample for comparison. This sample was simply rinsed with sterile PBS. After treating each sample accordingly, we performed gDNA extractions on the tergites exactly as we did for the experimental samples. We quantified the amount of resulting gDNA using a Nanodrop ND-1000 (Thermo Fisher Scientific, MA, USA). To screen for the presence of bacterial DNA specifically, we performed PCR on gDNA extractions using universal Bacterial primers. These PCR reactions contained 4.2uL nuclease-free water, 4uL 5PRIME HotMaster mix (Hilden, Germany), 0.5uL 25 mM MgCl_2_, 0.2uL forward primer 9 F, 0.2uL reverse primer 1046R, and 1uL of gDNA extraction product. Reactions were held at 94 °C for 2 min to denature the DNA, with 30 cycles of amplification proceeding at 94 °C for 20 s, 50 °C for 30 s, and 72 °C for 30 s, we added a final extension phase at 72 °C for 5 min, then cooled at 4 °C. Resulting amplicons were visualized on a 1 % agarose gel.

### Localization of AABs to the *C. chromaiodes* gut using diagnostic PCR

We developed a specific PCR screen for the presence of each AAB OTU that was detected through the microbiota characterization above. For this screen, we designed primers specific to each AAB OTU (AAB1 and AAB2). The 5′ primer coordinate for AAB1 corresponds to *E. coli* 16S position 828 (5′ TGGATGTCGGAGATTATGTCTTC 3′), which falls within hypervariable region 5. The corresponding 5′ coordinate for the AAB2 probe lies at position 628 (5′ GAAACTGCATTCAAGACGTGTAG 3′) and falls within hypervariable region 4. PCR reactions contained 10uL nuclease-free water, 10uL 5PRIME HotMaster mix (Hilden, Germany), 1uL DMSO, 1uL 25 mM MgCl2, 0.5uL of the AAB-specific forward primer, 0.5uL reverse primer 1046R, and 2uL of gDNA extraction product. Reaction conditions were as described above, but with a 54’C annealing temperature for 828 F and 58’C for 628 F.

We used this PCR screen to verify that AABs of local *C. chromaiodes* samples are located in the gut tract. After applying surface sterilization approach #2 (above) to whole ants, we dissected the entire gut tract (fore-, mid-, and hindgut tissue combined) of minor workers from multiple local colonies of *C. chromaiodes* under sterile conditions*,* extracted gDNA using the manufacturer’s protocol, and screened those samples as well as the tergite samples (described above) for the presence of each AAB OTU.

### Screening of additional *Camponotus* species and geographic regions

We used the described AAB primers to screen *Camponotus* ants of different species and various locations in the eastern and southwestern United States (Additional file 4). Samples were of two types: vouchers that were preserved in 70 % EtOH for up to seven years, and newly collected samples. For preserved voucher specimen, we used whole gasters that were surface sterilized under approach #2 (ethanol, bleach, and water rinses). For newly collected samples, within the same day of field collection, we surface sterilized whole ants using approach #2 and dissected the entire gut tract under sterile conditions. For both sample types, only one ant was used per sample. gDNA was prepared using the Qiagen (Hilden, Germany) DNEasy kit. PCR was performed as described above for the AAB-specific primers. All samples that yielded positive amplification with the specific primers described above were sequenced in both directions on an ABI 3730 xl DNA Analyzer (Thermo Fisher Scientific, MA, USA) by the Duke Genomic and Computational Biology Shared Resource. Sequence assembly was performed with Phrap [67]. Called bases were only accepted if they had a Q score >20 (99.9 % correct base call).

### Phylogenetic analysis of AAB 16S rDNA sequences

#### Generation of near full length 16S rDNA sequences

We generated near full length 16S rDNA sequences (spanning *E. coli c*oordinates 9–1507) for each AAB OTU using a combination of AAB-specific primers (above) and flanking universal primers. For AAB1, 16S sequences were generated for *Camponotus chromaiodes* (ID:793.2) and *Camponotus castaneus* (ID:854). For AAB2, 16S sequences were generated from *Camponotus chromaiodes* (799.2). To selectively amplify AAB1, we used forward primer 16S 828F (5′ TGGATGTCGGAGATTATGTCTTC ′3) and universal reverse primer 16S 1507R (5′ TACCTTGTTACGACTTCACCCCAG ′3). To amplify AAB2, we used 16S 628F (5′ GAAACTGCATTCAAGACGTGTAG ′3) and the same reverse primer, 16S 1507R. In order to generate the remainder of the 16S rDNA sequence, we used universal forward primer 16S 9F (5′ GAGTTTGATCCTGGCTCA ′3) and the reverse complement of the specific primers listed above. All products were sequenced on an ABI 3730xl (Life Technologies, Carlsbad, CA, USA).

#### Alignments

Bacterial 16S sequences were chosen from genera within the Acetobacteraceae that have host-associated species, as annotated by the RDP [38]. Sequence alignment was performed using SSU-ALIGN [68], a secondary structure-aware program based on the INFERNAL aligner [69]. Prior to phylogenetic analysis, all sequences were trimmed to a uniform length and regions were masked if the posterior probability of the nucleotide alignment fell below 0.95. Additionally, we masked all unaligned columns and removed any columns where more than 50 % of the sequences contained gaps. The final alignment spanned 1,248 positions of 16S rDNA across 53 taxa. This alignment is available as Additional file 9. This alignment was used to infer the broader tree of relationships (Fig. 1). Sequence divergences calculated from this alignment are likely conservative estimates of divergence, due to the trimming parameters described above.

Because relative rate analysis requires a well resolved tree, we generated a second Acetobacteraceae 16S rDNA alignment with fewer (36) taxa. Due to the decreased number of taxa, more alignment positions were unambiguous and thus could be included. This 36-taxa alignment includes 1,333 nucleotides, was generated with the same alignment parameters as described above, and is available as Additional file 10. This alignment was used to generate the rate analysis guide tree (Fig. 2), as detailed below.

#### Phylogenetic inference

For both alignments above, maximum likelihood and Bayesian approaches were used for phylogenetic inference. Maximum likelihood based inference employed RAxML [70], using a General Time Reversible (GTR) model with a Gamma (Γ) distribution of rate heterogeneity [71] and proportion of invariant site estimation (PInv). This model was chosen because jModelTest 2 [72] returned a nearly identical Bayesian information criterion (BIC) score to the top-scoring (Tamura-Nei) model and GTR models generally yield slightly better likelihood scores [73]. Bootstrap values were determined from 1000 replications.

MrBayes [74] was used for Bayesian phylogenetic inference, using a GTR + Γ + PInv model of nucleotide substitution, 10,000,000 generations, 4 chains, 1,000 generation sampling interval, and a 0.25 burn-in fraction. Genbank accession numbers for all taxa used in phylogenetic analyses are listed in Additional file 11.

#### Search of ribosomal database project

Near full length sequences from isolates of AAB1 and AAB2 (*E.coli* coordinates 9–1507) were aligned using RDP’s secondary-structure aware INFERNAL aligner [38]. Using the RDP SeqMatch tool, aligned sequences were queried against the entire database of 16S rRNA sequences, regardless of length, strain, or isolation source (3,224,600 sequences). The naïve Bayesian classifier was used for taxonomic assignment and trained on the latest training set (RDP Release 11, Update 4; training set 14). The latest search was performed on 15 March 2016. The RDP TreeBuilder tool was used to confirm that the most similar non-ant associated bacteria did not break up the monophyly of ant-associated AAB.

### Nucleotide substitution rate estimation

We used the baseml package within PAML4 [75] to estimate the nucleotide substitution rate of each AAB OTU at 16S rDNA. We used a GTR + Γ model of nucleotide substitution, as determined by jModelTest 2, based on the Bayesian information criterion [72]. The maximum likelihood tree of the 1,333 bp alignment (Fig. 2) was used as the guide in rate estimation. We chose to use this tree because it included enough taxa (*n* = 36) to robustly analyze rates across major lineages, was reasonably well resolved (ML bootstrap value >70) at most nodes, and had a congruent topology under ML and Bayesian inference (Fig. 2, Additional file 6).

We assessed the likelihood of various clock models under the GTR substitution model described above. To evaluate each clock scenario, we used PAML4 to calculate the likelihood score for that scenario (two or three distinct substitution rates) and used the likelihood ratio test (LRT) to calculate the p-value of that test. A more complex model (increased number of substitution rates) was accepted if it offered a significant improvement over the null hypothesis (a uniform rate).

### Ribosomal RNA folding energy estimation

We predicted the secondary structure and associated free energy estimation for each lineage listed in Additional file 7 using RNAfold [76]. We analyzed the near full length 16S rRNA sequence (*E. coli* coordinates 16–1477) of each OTU and constrained the alignment to the secondary structure of the 16S rRNA molecule. Due to expansion/deletion of various regions of 16S rRNA, direct comparisons of absolute free energy calculations were impossible. Therefore, we length-normalized free energy calculations to allow for cross-phyla comparisons. We constrained the predicted folding free energy of all molecules to empirically derived secondary structure interactions (based on the *E.coli* rRNA molecule*)*. We used the Turner 2004 model [77] to determine RNA folding free energies. This algorithm calculates both the partition function and base pairing probability matrix, in addition to the minimum free energy structure. We allowed dangling end energies on both sides of a helix to stabilize. All free energy calculations were assumed at a temperature of 37 °C.

## Abbreviations

AAB, acetic acid bacteria; gDNA, genomic DNA; GTR, general time reversible; OTU, operational taxonomic unit; PInv, proportion of invariant site estimation

## Additional files

Additional file 1: Methods and results of the estimation of empirical error rate for Ion PGM amplicon sequencing, based on an *E. coli* control library. (DOC 31 kb) Additional file 2: Empirical estimation of error rates for the *E. coli* amplicon library. (XLS 44 kb) Additional file 3: Sampling structure of collected *C. chromaiodes* colonies. (XLS 39 kb) Additional file 4: Distribution of AAB across ant samples. (XLS 50 kb) Additional file 5: Bootstrap consensus tree based on maximum likelihood analysis of Acetobacteraceae 16S rDNA. Phylogeny is based on the same 1,248 bp alignment that was used for Bayesian analysis shown in Fig. 1. The tree was reconstructed using a GTR + Γ model of nucleotide substitution. Node support was generated from 1,000 bootstrap resamplings. Branch lengths are unscaled, and do not reflect sequence distance. Environmental taxa are colored black, taxa associated with various insects are colored in orange, and the monophyletic ant AAB clade described here is colored green. *Roseomonas terrae* is the outgroup. (PDF 506 kb) Additional file 6: Bayesian phylogeny of Acetobacteraceae 16S rDNA. Phylogeny is based on the same 1,333 bp alignment that was used for maximum likelihood analysis shown in Fig. 2. A Markov chain Monte Carlo approximation was used for Bayesian inference of phylogenetic relationships. A GTR + Γ + PInv model of nucleotide substitution was implemented and the posterior probability of each node was estimated from 10,000,000 generations sampled in intervals of 1,000. Environmental taxa are colored black, taxa associated with various insects are colored in orange, and the monophyletic AAB clade described here is colored green. *Roseomonas terrae* is the outgroup. (PDF 21 kb) Additional file 7: Values for 16S rRNA stability and AT content, across free living and symbiotic bacteria shown in Fig. 3. (XLS 33 kb) Additional file 8: Primers used in this study. (XLS 29 kb) Additional file 9: Alignment file of 1,248 nucleotides and 53 taxa. This alignment was used for phylogenetic inference in Fig. 1 and Additional file 5. (TXT 66 kb) Additional file 10: Alignment file of 1,333 nucleotides and 36 taxa. This alignment was used for phylogenetic inference in Fig. 2 and Additional file 6. (TXT 48 kb) Additional file 11: Genbank accession numbers of all taxa used in phylogenetic analyses. (XLS 26 kb)
