# Supplementary material for: Deep divergence and rapid evolutionary rates in gut-associated Acetobacteraceae of ants
Source: BMC Microbiol. 2016 Jul 11;16:140. doi: 10.1186/s12866-016-0721-8 (PMC4939635; doi:10.1186/s12866-016-0721-8)
Supplement: Additional file 1: — Methods and results of the estimation of empirical error rate for Ion PGM amplicon sequencing, based on an E. coli control library. (DOC 31 kb) [file 12866_2016_721_MOESM1_ESM.doc]

**Methods and results of the estimation of empirical error rate for Ion PGM amplicon sequencing, based on an *E. coli* control library**

In light of concerns about high error rates for amplicon data generated on the Ion PGM [1], we determined the empirical error rate for Ion PGM amplicon sequencing by including a sample from a pure culture of the fully sequenced *E. coli* strain in our library construction and Ion PGM sequencing run.

**Methods:**

We used a pure culture from a single colony of *Escherichia coli* K12 substrain DH10B (TOP10) competent cells (Life Technologies), a line which has been fully sequenced [2], in order to determine sequencing error rates empirically. We prepared gDNA, digested the gDNA and resultant amplicons with PacI, created a barcoded library (as described above), and sequenced the library during the same sequencing run as all other samples. As detailed below (see *Variant analysis*), we then aligned reads against the known 16S rRNA sequence for this strain. We assessed the platform’s proclivity for indel and substitution errors and empirically calculated the per-base error rate. Any observed errors in the *E. coli* sample reflect those introduced during both PCR and Ion PGM sequencing, which was performed exactly as for our experimental (ant) samples.

All *E. coli* reads were stripped of barcodes and forward and reverse primers, and were aligned to the 16S rDNA gene of the K12 substrain DH10B [GenBank:CP000948] using the default parameters for –very-sensitive-local alignments in Bowtie2 [3]. Samtools was used to process all alignment files and create the variant-identified .pileup files [4]. Variants were analyzed using Varscan 2.2.3 [5].

**Results:**

Based on aligning all resulting *E. coli* reads >50 bp long (2,676 reads) to the known 16S rDNA sequence of this strain (one operon along V4-V5), we estimated the empirical error frequency at 0.00061 base substitution errors per site, and 0.006 indel errors per site (Additional file 2). We evaluated the frequency of both error types (base substitutions and indels) across reads. While most errors were unique, some errors were abundant in the dataset, with a maximal read frequency of 0.022 for a given substitution error and a maximal read frequency of 0.624 for a given indel error. This high incidence likely reflects a systematic error profile inherent to Ion Torrent pyrosequencing, as previously suggested [1].

We found that length-based thresholds (universal trimming of reads to 360 bp) followed by run-specific expected error filtering [6] proved to be the most effective way to reduce sequencing errors. In addition to mitigating OTU inflation derived from terminal alignment gaps [6], this strategy truncates the section of pyrosequencing reads with the highest error incidence [7]. For 400bp Ion PGM data, a precipitous increase in error rate characteristically occurs around flow 380 [8]. Accordingly, our approach reduced the total per-base error rate by an order of magnitude as compared to more standard length- and average Q score-based filtering strategies, while retaining up to 4-fold more reads than said strategies (Additional file 2). Our parameters for error filtering (1.5 expected errors per read) were experiment-specific and selected on the ability to maximize reads and successfully call a single OTU in the *E. coli* dataset.

**References**

1. Bragg LM, Stone G, Butler MK, Hugenholtz P, Tyson GW. Shining a light on dark sequencing: Characterising errors in Ion Torrent PGM data. PLoS Comput Biol. 2013; 9:e1003031.

2. Durfee T, Nelson R, Baldwin S, Plunkett G, 3rd, Burland V, Mau B, Petrosino JF, Qin X, Muzny DM, Ayele M *et al*. The complete genome sequence of escherichia coli dh10b: Insights into the biology of a laboratory workhorse. J Bacteriol. 2008; 190:2597-2606.

3. Langmead B, Salzberg SL. Fast gapped-read alignment with bowtie 2. Nat Methods. 2012; 9:357-359.

4. Li H, Handsaker B, Wysoker A, Fennell T, Ruan J, Homer N, Marth G, Abecasis G, Durbin R, Genome Project Data Processing S. The sequence alignment/map format and samtools. Bioinformatics. 2009; 25:2078-2079.

5. Koboldt DC, Zhang Q, Larson DE, Shen D, McLellan MD, Lin L, Miller CA, Mardis ER, Ding L, Wilson RK. Varscan 2: Somatic mutation and copy number alteration discovery in cancer by exome sequencing. Genome Res. 2012; 22:568-576.

6. Edgar RC. Uparse: Highly accurate OTU sequences from microbial amplicon reads. Nat Methods. 2013; 10:996-998.

7. Huse SM, Huber JA, Morrison HG, Sogin ML, Welch DM. Accuracy and quality of massively parallel DNA pyrosequencing. Genome Biol. 2007; 8:R143.

8. Golan D, Medvedev P. Using state machines to model the Ion Torrent sequencing process and to improve read error rates. Bioinformatics. 2013; 29:i344-i351
