# Supplementary figures and images for: Deep divergence and rapid evolutionary rates in gut-associated Acetobacteraceae of ants
Source: BMC Microbiol. 2016 Jul 11;16:140. doi: 10.1186/s12866-016-0721-8 (PMC4939635; doi:10.1186/s12866-016-0721-8)

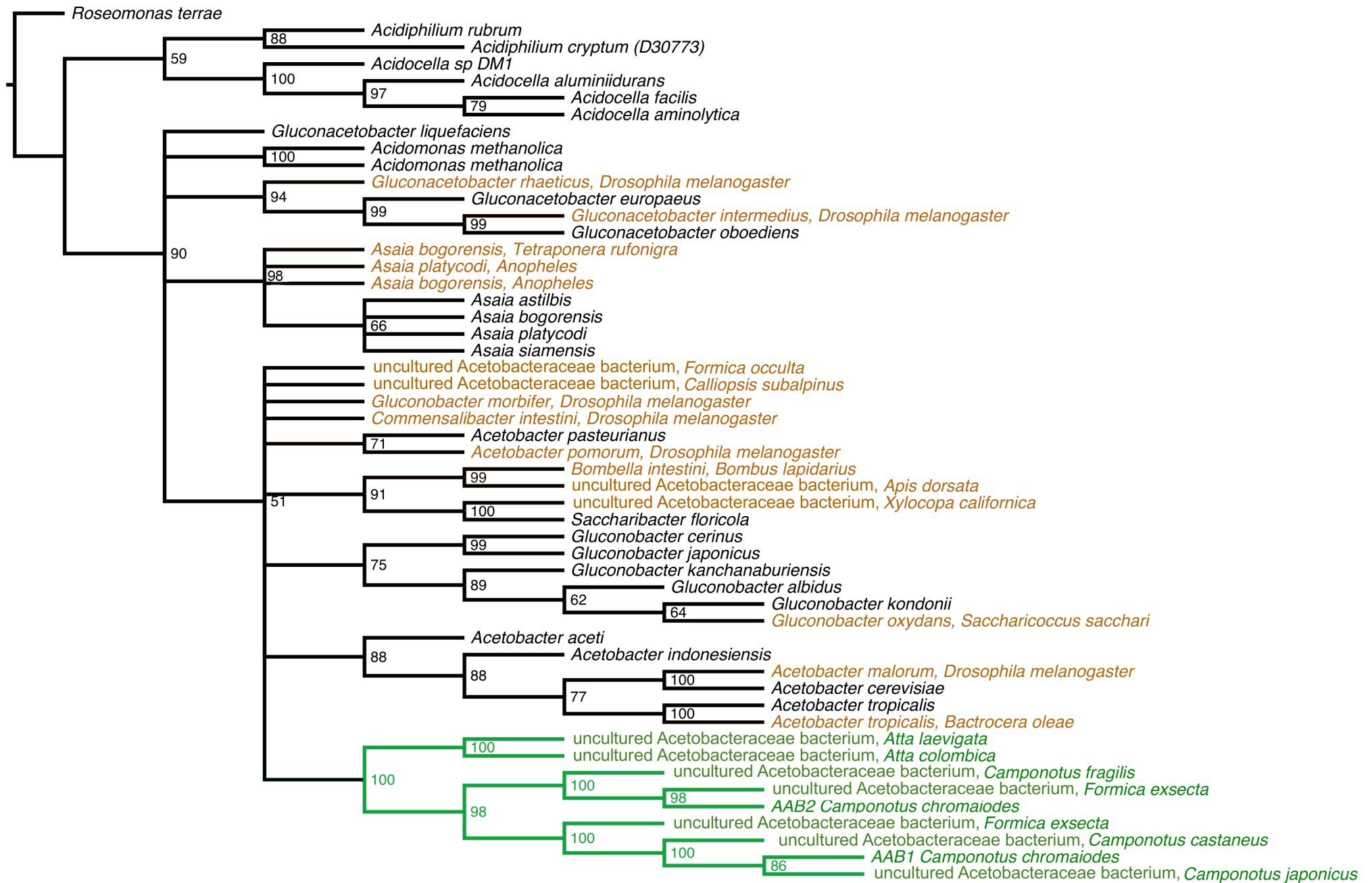

Supplement: Additional file 5: — Bootstrap consensus tree based on maximum likelihood analysis of Acetobacteraceae 16S rDNA. Phylogeny is based on the same 1,248 bp alignment that was used for Bayesian analysis shown in Fig. 1. The tree was reconstructed using a GTR + Γ model of nucleotide substitution. Node support was generated from 1,000 bootstrap resamplings. Branch lengths are unscaled, and do not reflect sequence distance. Environmental taxa are colored black, taxa associated with various insects are colored in orange, and the monophyletic ant AAB clade described here is colored green. Roseomonas terrae is the outgroup. (PDF 506 kb) [file 12866_2016_721_MOESM5_ESM.pdf]

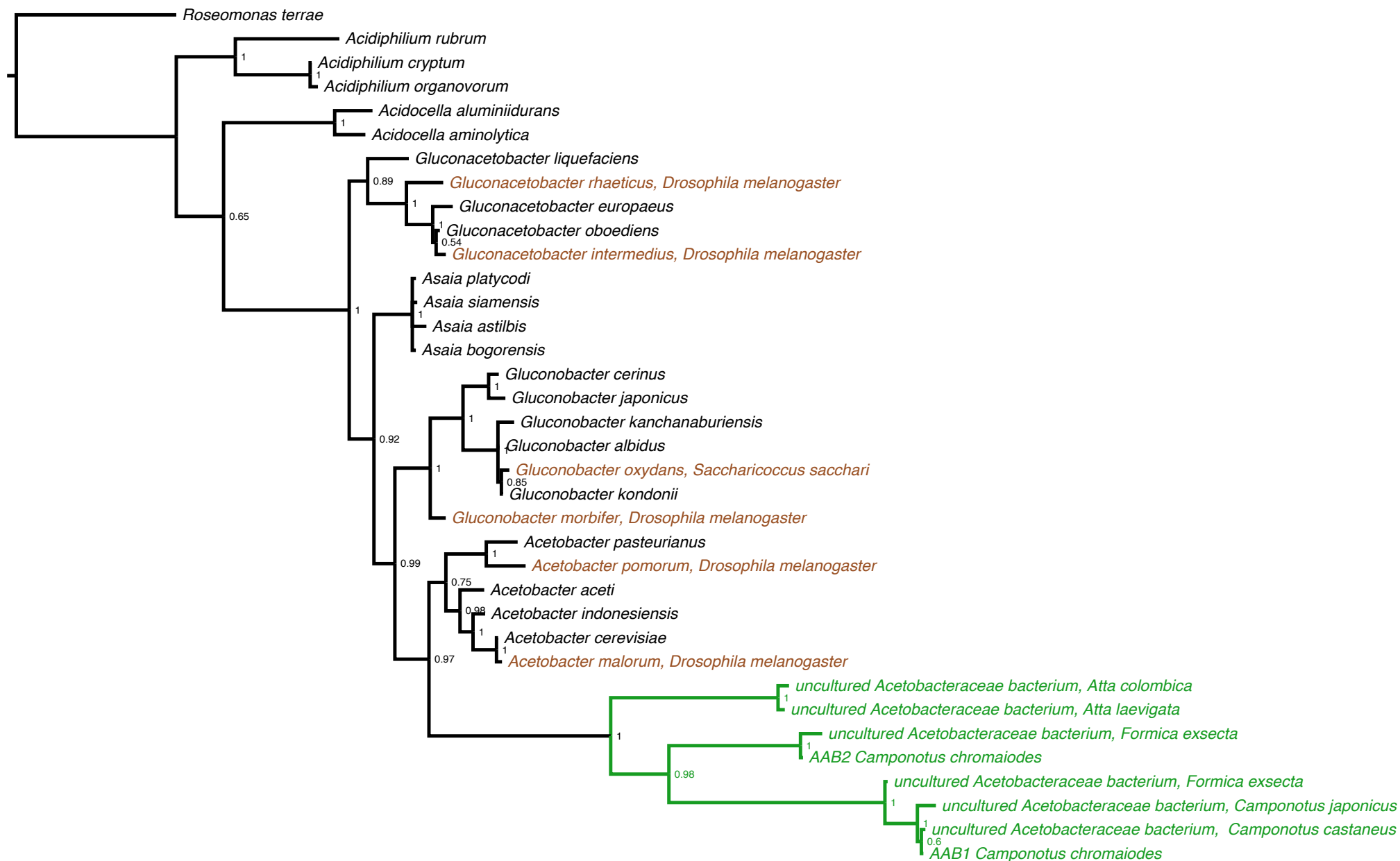

0.3

Supplement: Additional file 6: — Bayesian phylogeny of Acetobacteraceae 16S rDNA. Phylogeny is based on the same 1,333 bp alignment that was used for maximum likelihood analysis shown in Fig. 2. A Markov chain Monte Carlo approximation was used for Bayesian inference of phylogenetic relationships. A GTR + Γ + PInv model of nucleotide substitution was implemented and the posterior probability of each node was estimated from 10,000,000 generations sampled in intervals of 1,000. Environmental taxa are colored black, taxa associated with various insects are colored in orange, and the monophyletic AAB clade described here is colored green. Roseomonas terrae is the outgroup. (PDF 21 kb) [file 12866_2016_721_MOESM6_ESM.pdf]
